# CD30 Is Highly Expressed in Chronic Obstructive Pulmonary Disease and Induces the Pulmonary Vascular Remodeling

**DOI:** 10.1155/2018/3261436

**Published:** 2018-06-10

**Authors:** Liang Luo, Yangli Liu, Dubo Chen, Fengjia Chen, Hai Bing Lan, Canmao Xie

**Affiliations:** ^1^The Division of Pulmonary and Critical Care Medicine, The First Affiliated Hospital of Sun Yat-sen University, 58 Zhongshan Road, Guangzhou, Guangdong Province, China; ^2^Department of Critical Care Medicine, The Seventh Affiliated Hospital of Sun Yat-sen University, 628 Zhenyuan Road, Shenzhen, Guangdong Province, China; ^3^Laboratory Medicine, The First Affiliated Hospital of Sun Yat-sen University, 58 Zhongshan Road, Guangzhou, Guangdong Province, China; ^4^Respiratory Department, The Second Affiliated Hospital of NanChang University, No. 1 Min De Road, NanChang, JiangXi Province, China

## Abstract

Chronic obstructive pulmonary disease (COPD) is one of the common and underdiagnosed diseases with the highest morbidity and mortality in the world. The development of COPD can lead to pulmonary vascular remodeling and pulmonary hypertension, further causing the occurrence of pulmonary heart disease. Therefore, attenuation of pulmonary vascular remodeling and pulmonary hypertension caused by COPD can significantly delay cardiovascular complications. In the study, we firstly found that the expression of CD30 and CD30L was increased in COPD. Importantly, the serum CD30L levels were significantly higher in patients with stable COPD relative to those with acute exacerbation of COPD (AECOPD). This suggested that CD30 might be related to the development of COPD. In addition, we found that the expression of CD30 in the COPD rat model was significantly increased compared with control group. And treatment with the anti-CD30 antibody reduced the serum concentration and tissue expression of CD30 in rat. Importantly, anti-CD30 antibody alleviated pulmonary vascular remodeling in COPD model rats. This suggested that CD30 played an important role in the course of COPD. Finally, we found that, in the HPASMC and HPAEC cell lines, CD30 can affect the cell viability and cell migration and inhibited hypoxia-induced cell apoptosis in a concentration-dependent manner. We also found CD30 induced extracellular matrix formation through decreasing the expression of MMP-2, thus promoting the pulmonary vascular remodeling. The study indicated that CD30 and CD30L were involved in pulmonary vascular remodeling and inflammatory response in COPD. Altogether, CD30 might be a marker for the early diagnosis and progression of COPD.

## 1. Introduction

Chronic obstructive pulmonary disease (COPD) is a common disease around the world [1] that exerts a high morbidity in the middle-aged and elderly population. With the increase of the proportion of smoking population and the aggravation of environmental pollution, the morbidity and mortality of COPD are increased year by year. The Global Burden of Disease Study speculates that COPD will be the third cause of human death by 2020. In addition, the WHO shows that the human burden for medical treatment for COPD will be the fifth highest by 2020 [2, 3]. The chronic hypoxia, hypercapnia, and acidosis can lead to pulmonary hypertension by causing extensive contraction of pulmonary vascular remodeling in COPD patients. Chronic inflammation stimulation can also lead to vascular endothelium and smooth muscle hyperplasia and pulmonary vascular structural changes such as stenosis, occlusion, and fibrosis, resulting in further pulmonary vascular remodeling. At advanced stage of COPD, the most common cardiovascular complication is pulmonary hypertension, which further leads to chronic pulmonary heart disease, right heart failure, and even heart failure [4]. Recently, several studies have shown that pulmonary vascular remodeling and pulmonary arterial hypertension are the most critical aspects of pulmonary heart disease in COPD patients [5, 6]. Therefore, the remission of COPD pulmonary hypertension and pulmonary vascular remodeling in early stage can delay the occurrence of cardiovascular complications. Unfortunately, there are few targeted drugs for the treatment of pulmonary hypertension induced by COPD.

So far the pathogenesis of COPD still remains unclear. It is known that smoking, genetic factors, respiratory tract infection, and protease imbalance are closely related to the pathogenesis of COPD. Inhalation of gases or substances elicits oxidative stress in the lungs and destroys the balance between protease and antiprotease and eventually causes inflammation, one of the most important factors known for the pathogenesis of COPD. The inflammatory cells of COPD patients include monocytes, macrophages, and neutrophils. When the patients are stimulated by factors such as smoking and infection, the inflammatory cell is activated and releases cytokines rapidly. The cytokines further aggravate the inflammatory reaction, induce the synthesis of adhesion molecules in vascular endothelial cells, and then damage lung tissue structure [7]. In addition, dysfunction of the autonomic nervous system also plays a vital role in the development of COPD. In addition, decline in organism immunity leads to the invasion of some bacteria into the body and their multiplication in the body. These bacteria produce a large amount of internal and external toxin to damage lung tissue and induce inflammatory reaction and even systemic inflammatory response [8].

Previous study suggests that the activated CD4^+^ T cells in the alveolar epithelium of COPD secrete large amounts of TNF-a, IL-6, MMP-9, and other inflammatory mediators and then destroy the extracellular matrix and thereby lead to emphysema [9, 10]. CD30, a member of the tumor necrosis factor receptor superfamily, also known as Ki-1 [11], is a kind of type I transmembrane glycoprotein. The CD30 protein is composed of 595 amino acids, and the cytosolic and transmembrane region in the protein are composed of 188 amino acids and 24 amino acids, respectively. The extracellular region is the leading peptide of 18 amino acids, followed by 365 amino acids. Under certain conditions, MMPs can cut off the extracellular region of the protein and form soluble CD30 (sCD30), with relative molecular mass 85KD. CD30L, another member of the tumor necrosis factor superfamily, is a type II transmembrane glycoprotein with a molecular mass of 40 KD. CD30L (TNFSF8) is a 26–40 kD type II membrane glycoprotein belonging to the 148 TNFSF. CD30L protein has a cytosolic region consisting of 40 amino acid residues at the ammonia end, with a cytosolic outer zone consisting of 172 amino acids at the carbon terminal. In general, CD30L exists as trimer and the extracellular region is homologous to TNF-a, TNF-*β*, and CD40L [12–16].

CD30 is widely expressed in viral-infected lymphocytes and lymphoma cells and activated T cells. Moreover, CD30 is also expressed in alveolar macrophages, epidermal cells, activated NK cells, decidual cells, resting B cells, granulocytes, thymocytes, and medulla cells [17]. Younes et al. found that the sCD30 in serum can bind to CD30L on peripheral blood lymphocytes, which leads to disruption of cell interaction and thereby cutting off signal transduction [16]. Many studies have shown that CD30 and CD30L play a vital role in signal transduction in cells. The bind of CD30 and CD30L induces the production of cytokine, which promotes cell proliferation, differentiation, cell growth, stagnation, and death [18].

The levels of sCD30 in serum are very low in healthy adults, but sCD30 levels are markedly increased in serum or body fluids in some pathological conditions [19]. Del Prete et al. found that CD30 has more expression in Th2 cells in patients with asthma, measles, Omenn syndrome, and other diseases dominated by the Th2 reaction, and CD30+ cells and sCD30 levels in patients with peripheral blood circulation in the proportion increased [20]. In some diseases caused by Th1 reactions such as tuberculosis and Wegener's granuloma, the number of CD30+ cells in the blood circulation is increased and positively related to the amount of serum sCD30. In addition, they also found that CD30+ cells can produce INF-*γ* [21]. Pellegrini et al. have confirmed that CD30 is a potential regulator of the balance between Th1 and Th2 and expressed in both Th2 and Th1 cells [22].

Some clinical studies have demonstrated that in some malignant tumors, autoimmune diseases, and infectious diseases, the content of sCD30/CD30L is increased in serum. These results suggest sCD30/CD30L in serum can be used as a marker for diagnosis and prognosis of these diseases. For example, by detecting the serum concentration of CD30, the severity of bronchial asthma can be indirectly predicted. The inflammatory reaction and COPD asthma have certain similarity. Therefore, we speculate that CD30 and CD30L can be associated with occurrence and development of COPD disease. Notably, there is no report about the correlation between CD30/CD30L and COPD.

Recent studies have shown that pulmonary hypertension and pulmonary vascular remodeling are the most important steps in the development of COPD in patients with pulmonary heart disease. Therefore, the attenuation of pulmonary vascular remodeling and pulmonary hypertension by effective control of COPD in the early stage of disease is important for delaying its cardiovascular complications. In this study we showed that the CD30/CD30L was positively related to pulmonary vascular remodeling and inflammatory reaction in COPD. CD30/CD30L was associated with the pulmonary vascular remodeling and inflammation in the process. Our results showed CD30/CD30L could be used as a marker for inflammation of COPD and pulmonary vascular remodeling.

## 2. Materials and Methods

### 2.1. Participants

The study included a total of 106 patients, who were recruited from the First Affiliated Hospital of Sun Yat-sen University, from March 2014 to July 2015. These patients included 37 COPD patients, 42 acute exacerbations of COPD (AECOPD) patients, and 27 healthy individuals. The diagnosis and treatment of COPD/AECOPD patients were based on guidelines for the diagnosis and treatment of COPD. Stable phase COPD patients recovered at least 8 weeks after the last illness. This study was approved by the Ethics Committee of the First Affiliated Hospital of Sun Yat-sen University and all aspects of the study complied with the Declaration of Helsinki. All the subjects in this study signed the informed consent for participating in this study.

*Inclusion Criteria*

1. more than 18 years of age;
2. complete case history data and clinical, laboratory, and ancillary examination results;
3. healthy adults who had no history of chronic disease as a healthy control group.

*Exclusion Criteria*

1. with other chronic lung disease patients, including interstitial lung disease and bronchial asthma;
2. with malignant tumor patients;
3. with autoimmune diseases.

### 2.2. Physician Survey

Pulmonary function test: the pulmonary function instrument (MasterScreen Diffusion, Jaeger, Germany) was used to measure FEV1, FEV1, and FEV1/FVC. Cardiac ultrasonography: the heart function and the heart rate of patients in stable COPD group and AECOPD group were detected by heart color Doppler diagnostic apparatus (iE33, Philips, Holland) and LVEF and PASP values were recorded. Arterial blood gas analysis: PO2, PCO2, and blood oxygen saturation were recorded through arterial blood gas analysis.

### 2.3. ELISA

The proteins and cytokine in serum and cell supernatant were determined by enzyme-linked immunosorbent assay (ELISA), following the kit instructions strictly. Readings were performed on a full-wavelength microplate reader (SpectraMax Plus384, USA). CD30 and CD30L ELISA kits were purchased from Shanghai FANKEL industrial Co., Ltd. VEGF, MMP-9, MMP-2, and ECM1 ELISA were purchased from Cloud-Clone Corp. (Wu Han, China).

### 2.4. Construction of a Rat Model of COPD

Sprague-Dawley (SD) rats were purchased from Shanghai SLAC Laboratory Animal Co., Ltd. All procedures were approved by Sun Yat-sen University Clinic Institutional Animal Care and Use Committee. The animals were raised for 6 days at a temperature of 20~25°C and humidity within 40–70% range. SD rats were randomly divided into normal control group, COPD control group, and COPD anti-CD30 antibody intervention group, with 5 rats in each group. The COPD model is established by combined cigarette smoke and tracheal instillation of LPS [23–26]. In brief, the SD rats are placed in a smoking exposure box, and the size of the smoke exposure box is about 65 cm × 50 cm × 35 cm, a diameter 5 cm smoke exhaust opening is provided on each side, and a 10 cm inlet is opened below. Keeping the smoke state, each rat passively inhaled 6 to 8 cigarettes, two times a day, 30 minutes at a time with an intervals of 5~6 hours, continuously for 59 days. 10% chloral hydrate at a dose of 3 ml/kg rats was anesthetized by intraperitoneal injection, and the rats were supinely fixed on the operating table, a longitudinal incision of the neck skin was done, exposing the infrahyoid muscles, and then the trachea was isolated. The slow infusion concentration was 1 mg/ml LPS, each rat was injected with 0.2 ml, and then the rats were put up to make LPS flow evenly into the lung and then suture the incision. LPS was purchased from Sigma company (item number: LOT 7391 × 15 V) before use, stored away from light at 4°C, when using saline with a final concentration of 1 mg/ml solution configuration. Rats were injected with lipopolysaccharide (LPS) 0.2 ml (200 mg) (airway drip test) for first days and twenty-eighth days, and no smoking was allowed within that day. The normal control group at the same time was allowed to breathe in smoking exposure box homemade in normal air, and, on the first day and the twenty-eighth day, the 0.2 mL of normal saline in the same way drops into the normal control group rats airway. After the successful construction of the COPD rats model, 150 *μ*g anti-CD30 antibody was intraperitoneally injected 2 times a week for 4 weeks in the anti-CD30 model group while the same dose of normal saline was intraperitoneally injected in COPD model group and the control group. The mortality of the rats was zero.

### 2.5. HE Staining and Immunohistochemical Analysis

The protocol of HE staining followed a previous study [27]. Immunohistochemistry was used to detect the level of protein expression in rat tissue sections. Immunohistochemistry was performed as previously described [28].

CD30 antibody from Bioss Antibodies (Beijing, China), CD30L antibody purchased from Boster Biological Technology (Wu Han, China). VEGF and MMP-9 antibody were purchased from Abcam; rabbit secondary antibody (K152411G) and DAB kit were purchased from Beijing Zhongshan Golden Bridge Biotechnology Co. Ltd. Under the microscope (BX43, Olympus) three different horizons (200x) were sliced on each piece of immunohistochemical observation. The results were scored on a 5-tiered scale: 0, 0% positive cells or no staining; 1+, <25% positive cells; 2+, 25–50% positive cells; 3+, >50–75% positive cells; and 4+, >75% positive cells. The staining intensity of each field of view was scored, 0 of which were less marked, 1 point for slight staining, 2 points for moderate, and 3 points for severe staining. The final score was positive for cell percentage and staining intensity and 0~1 for negative, 1~4 for weak positive, 4~6 for moderately positive, and 6~7 for strong positive.

### 2.6. Vascular Diameter Determination

Vascular diameter determination in rat models was measured by Image-Pro Plus software (Media Cybernetics, USA).

### 2.7. Western Blot Analysis

Cells were collected and lysed on ice in cell lysis buffer (Beyotime Institute of Biotechnology, China). The protein concentration was determined by Bradford protein assay (Beyotime Institute of Biotechnology, China). Equal amounts (20 to 40 *μ*g) of cell extract were subjected to electrophoresis in 10–12% sodium dodecylsulfate-polyacrylamide (SDS-PAGE) and transferred to PVDF membranes (Millipore, USA) for Western blotting. The membranes were blocked with 5% skim milk powder and 0.1% Tween-20 in PBS for 1 h at room temperature and then incubated with specific primary hypoxia-inducible factor-1 alpha (HIF-1*α*) antibody (Abcam, USA). After being washed by PBS with 0.1% Tween-20 for three times, the membrane was incubated with a HRP-conjugated secondary antibody at room temperature for 1 h. GAPDH (Abcam, USA) was used as the internal protein. The membranes were then visualized using BeyoECL Plus Kit (Beyotime Institute of Biotechnology, China) according to the manufacturer's instructions and photographed by the ChemiDoc XRS gel documentation system (Bio-Rad, Hercules, USA).

### 2.8. Cell Viability, Transwell Migration, and Apoptosis Assays

Cell viability was analyzed by a standard CCK8 assay (Dojindo, Japan).

Transwell migration was performed for detecting the cell migration ability as previously described [29]. Cell apoptosis was detected by using the Apoptosis Detection Kit (Multisciences, China) following the manufacturer's protocol as described previously [30].

### 2.9. Statistical Analysis

Student's t-tests were conducted using the Statistical Package for Social Sciences (SPSS) software (version 19.0) and P value < 0.05 was considered significant. Every experiment in this study was performed for three times and data were presented as mean ± standard deviation.

## 3. Results

### 3.1. Comparison of the Clinical Data between Participants

As shown in Table 1, there were significant differences in age and smoking between COPD group and AECOPD group (P < 0.05). The ages were significantly higher in AECOPD group than those in COPD group. The frequency of smoking was significantly higher in AECOPD group than that of COPD group. These data suggested that acute exacerbations in COPD were closely related to age and smoking and that the likelihood of exacerbations increases with age or smoking.

There was no significant difference between the two groups in oxygenation index (PaO_2_/FiO_2_). But the PaCO_2_ was significantly higher in AECOPD group than in the COPD group (P < 0.0001). Pulmonary ventilation function index FEV1 (L) (forced expiratory volume) and FEV1/FVC (%) were significantly decreased in the AECOPD group than in the COPD group. There was no significant difference in LVEF (%) between the two groups. It conforms to the pathophysiological features of acute exacerbation of COPD. But the heart rate index of the AECOPD group was significantly higher than that of the COPD group (P < 0.0001). And PASP (mmHg) index was higher in AECOPD group than the COPD group (P < 0.05). It may be related to chronic hypoxia, long-term storage of carbon dioxide, stimulation of pulmonary vessels and pulmonary vascular bed, reduction of pulmonary vascular obstruction, inflammation, and accumulation of blood and sodium in the blood.

### 3.2. sCD30 and CD30L Levels Were Upregulated in the Stable COPD Patients

**Figure 1:**
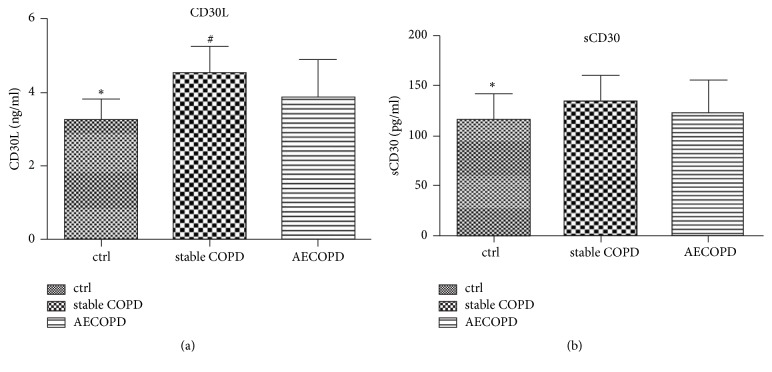
*The level of CD30 and CD30L in serum from the patients with stable COPD and AECOPD. *((a) and (b)) The level of CD30L and sCD30 in serum from stable COPD and AECOPD and the normal group. Columns, mean of the group; bars, SD. ^*∗*^*P* < 0.05, normal group versus AECOPD group. ^#^*P* < 0.05, stable COPD group versus AECOPD group.

To evaluate the role of CD30 in COPD, the levels of CD30 and CD30L were measured in the stable COPD, AECOPD, and normal group, respectively. Results showed that the level of CD30L was 4.52 ± 0.75, 3.88 ± 1.04, and 3.25 ± 0.58 ng/ml in the stable COPD, AECOPD, and normal group, respectively. As shown in Figure 1(a), comparing with the normal group, the level of CD30L was statistically significantly upregulated in the stable COPD and AECOPD group (P < 0.05). The level of CD30L was the highest in COPD group and the lowest in the control group. The level of CD30L was 116.54 ± 25.66 pg/ml in normal group, 134.80 ± 25.21 pg/ml in stable COPD group, and 123.82 ± 32.31 pg/ml in AECOPD group. The level of sCD30 was significantly downregulated in the normal group compared with stable COPD group (P < 0.05). The level of sCD30 exerted no significant difference between the normal group and the AECOPD group (Figure 1(b)). In addition, there is no statistical difference between AECOPD and COPD group. These results suggested that the level of CD30L and CD30 can reflect the difference between COPD patients and normal population.

### 3.3. CD30 and CD30L Are Positively Correlated with Pulmonary Vascular Remodeling in COPD Rat Model

**Figure 2:**
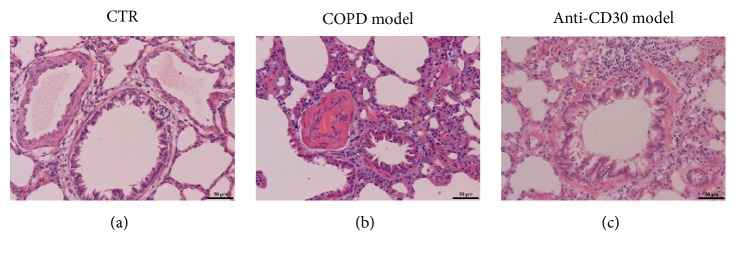
*Lung tissue structure characteristics in rat models. *Representative images of lung tissue structure were determined by HE staining in control group (a), COPD model group (b), and anti-CD30 treated COPD model group (c). HE ×200.

To investigate the roles of CD30 and CD30L in COPD, pulmonary vascular remodeling, and inflammatory response, we constructed the COPD rat model. We got the lower lobe of right lung from all the model groups by the operating. And HE staining was performed to observe the lung tissue structure. As shown in Figure 2(a), there appeared normal lung tissue, clear alveolar in the control group; however inflammation was not observed in control group. However, in the COPD group, the infiltrations of inflammatory cells were observed in the airway and vessels, the alveolar walls were thick, and pulmonary hemorrhage and pulmonary emphysema were observed (Figure 2(b)). In the anti-CD30 antibody treatment group, the inflammatory cells were also observed, but no hemorrhage and emphysema. The results suggested that the rat model of COPD was constructed successfully.

### 3.4. Detection of Vascular Remodeling Index in Lung Tissue of Rat Model

To explore the role of CD30 in the COPD and pulmonary vascular remodeling, vascular area/total vascular area (WA%) and vascular wall thickness (WT) were measured. As shown in the Table 2, WA% and WT vessels between 0 and 50 *μ*m in the control group were 56%  ± 8% and 6.96 ± 1.93 *μ*m, respectively. In the COPD model group, WA% and WT vessels within 0–50 *μ*m were 65%  ± 14% and 7.34 ± 2.45 *μ*m, respectively. In the anti-CD30 COPD model group, WA% and WT vessels within 0–50 *μ*m were 65%  ± 29% and 7.63 ± 2.46 *μ*m, respectively. WA% and WT in the normal group were lower relative to the model group and anti-CD30 group (P < 0.05). There was no remarkable difference between the model group and the anti-CD30 group. In the scope of 50–90 *μ*m, WA% and WT vessels in the COPD model group were highest in these three groups (P < 0.05). WA% and WT vessels in the control group were higher than the anti-CD30L group (P < 0.05). In the scope of 90–200 *μ*m, WA% and WT vessels in the COPD model group were highest in these three groups (P < 0.05). And there was no remarkable difference between the control group and the anti-CD30 group. The results showed a significant vascular remodeling in the COPD model group. However, the anti-CD30 antibody treatment prevented vascular remodeling.

### 3.5. Immunohistochemical Analysis of CD30, CD30L, MMP-9, and VEGF Expression

**Figure 3:**
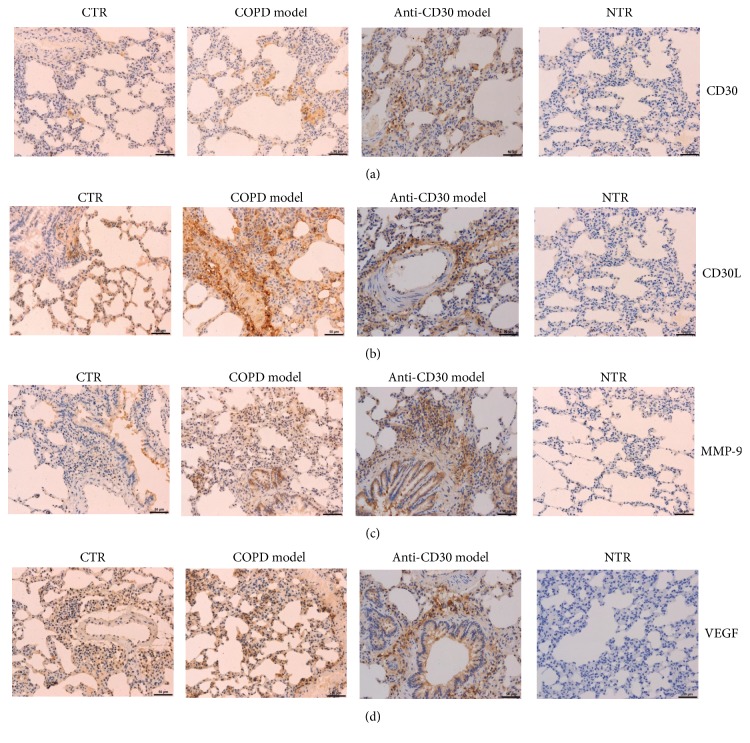
*Immunohistochemical analysis of CD30, CD30L, MMP-9, and VEGF expression in rat models. *(A) The expression levels of CD30, CD30L, MMP-9, and VEGF in lung tissues of rat model groups were determined by IHC. CTR: control group; NTR: negative control group. IHC ×200.

Based on the aforementioned scoring system, scores of CD30 expression in control group, COPD model group, and anti-CD30L COPD group were 3.07 ± 0.46, 5.78 ± 0.55, and 5.42 ± 0.90, respectively (Figure 3(a)). Scores of CD30L expression in control group, COPD model group, and anti-CD30L COPD group were 2.80 ± 0.68, 6.33 ± 0.49, and 5.25 ± 0.62, respectively (Figure 3(b)). Score of MMP-9 expression in the control group was 3.33 ± 0.49, which is lower than that in COPD model group and anti-CD30 model group. And then there were no obvious different scores between COPD model group and anti-CD30 model group with 5.17 ± 0.71 and 5.08 ± 0.99, respectively (Figure 3(c)). The score of VEGF in COPD model group with 5.67 ± 0.49 was higher than that in control group with 4.07 ± 0.26. And there were no remarkable differences compared with anti-CD30 model group with score of 4.83 ± 0.72 (Figure 3(d)).

Correlation analysis indicated that the expressions of inflammatory factors CD30 and CD30L in COPD were positively correlated to the VEGF and MMP-9. The total score of the staining intensity and the percentage of positive cells of CD30, CD30L, VEGF, and MMP-9 were increased synchronously. With the intervention of CD30 antibody, the level of CD30 and CD30L decreased significantly both in the serum and in the tissue, and it improved the pulmonary vascular modeling. This implied that CD30 was an inflammatory factor in the occurrence and development of COPD.

### 3.6. Effect of CD30 on the Function of HPAEC and HPASMC Cell Lines

**Figure 4:**
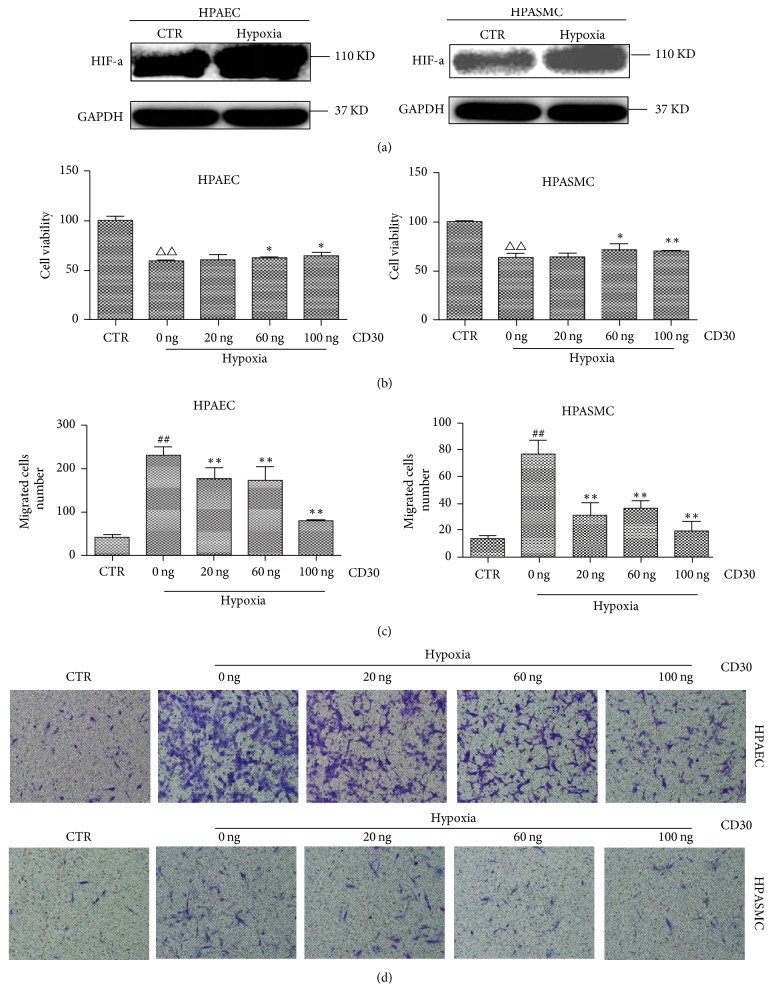
*CD30 decreased cell viability and cell migration in HPAEC and HPASMC cells. *(a) Hypoxia induced the HIF-a expression in HPAEC and HPASMC cell lines. The proteins levels were detected by Western blotting. GAPDH as the internal control. (b) Cell viability was detected in HPAEC and HPASMC cell lines. CTR: control cells. Hypoxia: cells were cultured under anoxic conditions (oxygen concentration: 10 ± 0.5% O2) for 24 h. Hypoxia-group cells were treated by 20 ng, 60 ng, and 100 ng of CD30, respectively, for 24 h. △△ compared with control group cells, *P* < 0.01; *∗* and *∗∗* compared with hypoxia-group cells, *P* < 0.05 and *P* < 0.01, respectively. ((c) and (d)) Cell migration viability was detected by transwell assays in HPAEC and HPASMC cell lines. ## compared with control group cells, *P* < 0.01.  *∗* compared with hypoxia-group cells, *P* < 0.05. *∗∗* compared with hypoxia-group cells, *P* < 0.01.

Previous studies have demonstrated that hypoxia promoted cell proliferation in HPASMC and induced abnormal apoptosis and proliferation in HPAEC cells, therefore leading to pulmonary vascular remodeling and pulmonary arterial hypertension [31–33]. To further investigate the role of CD30 in pulmonary vascular remodeling, HPAEC and HPASMC cell lines were used in the study. HPAEC and HPASMC were cultured under anoxic conditions for 24 h. Subsequently, Western blotting was performed to detect the expression of HIF-1a in HPAEC and HPASMC cell lines. As shown in Figure 4(a), the expression of HIF-1a was increased obviously in hypoxic compared with control cell. It indicated that the hypoxic cell model was successfully established. However, the level of reactive oxygen species (ROS) in cells was not obviously changed (data not shown). Then, cell viability was detected by CCK8 assay. As shown in Figure 4(b), the cell viability in hypoxia-HPAEC cells was lower than the control cell (P < 0.01). After treatment by 60 ng/ml and 100 ng/ml CD30, we found CD30 significantly increased cell viability in dose-dependent manner in HPAEC cell under hypoxic conditions (Figure 4(b), left). Similarly, CD30 increased the cell viability in dose-dependent manner in HPASMC cell under hypoxic conditions. The above results suggested that CD30 can block cell damage induced by hypoxia in HPAEC and HPASMC cells. These results also suggested CD30 can promote pulmonary vascular remodeling through stimulating the growth of HPAEC and HPASMC cells.

**Figure 5:**
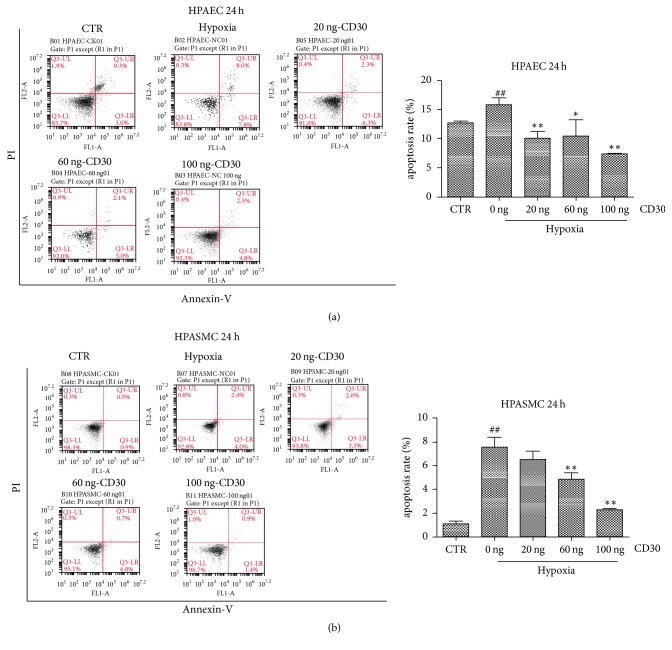
*CD30 inhibited cell apoptosis induced by hypoxia in HPAEC and HPASMC cells.* ((a) and (b)) Flow cytometry analysis of the cell apoptosis in HPAEC and HPASMC cells. CTR: control cells. Hypoxia: cells were cultured under anoxic conditions (10 ± 0.5% O_2_) for 24 h. ## compared with control group cells, *P* < 0.01.  *∗* compared with hypoxia-group cells, *P* < 0.05. *∗∗* compared with hypoxia-group cells, *P* < 0.01.

Cell migration of vascular endothelial cells plays an important role in the occurrence and development of pulmonary artery remodeling [29]. Transwell assay was performed for detection of the ability of cell migration. As shown in Figures 4(c) and 4(d), hypoxia increased cell migration in HPAEC and HPASMC cells (P < 0.01). However, CD30 recombinant protein decreased the migration ability in concentration-dependent manner in the two cells under hypoxic condition. Next, we detected the effect of CD30 on apoptosis in HPAEC and HPASMC cell lines. The apoptosis assay showed that hypoxia increased the cell apoptosis rate in the two cells. Moreover, we showed CD30 decreased cell apoptosis in a concentration-dependent manner in the two cells (Figure 5). This suggested that CD30 inhibited hypoxia-induced apoptosis in HPASMC and HPAEC cells, thereby affecting pulmonary vascular remodeling.

**Figure 6:**
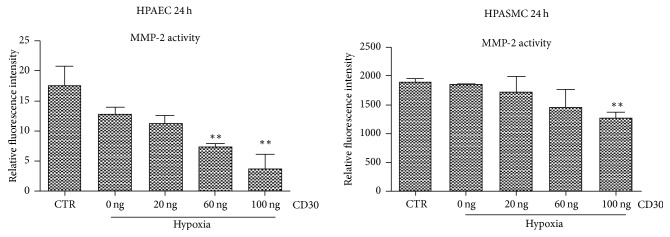
*CD30 decreased the level of MMP-2 in the HPAEC and HPASMC cells. *HPAEC and HPASMC cells were treated with indicated dose of CD30. And then MMP2 activities were detected as described in Materials and Methods in the cell supernatant. Relative fluorescence intensity indicated the activities of MMP-2. CTR: control cells. Hypoxia: cells were cultured under anoxic conditions (10 ± 0.5% O_2_) for 24 h. *∗∗* compared with hypoxia-group cells, *P* < 0.01.

To further investigate the role of CD30 in pulmonary vascular remodeling, ELISA assay was performed to detect the cell factors in cell culture medium. The results showed that the level of MMP-2 was obviously decreased by CD30 in HPAEC and HPASMC cell line (P < 0.01) (Figure 6). These results suggested CD30 affects the pulmonary vascular remodeling by reducing the expression of MMP-2 and regulating the formation of extracellular matrix under hypoxia in HPAEC and HPASMC cells.

## 4. Discussion

COPD is a disease characterized by airflow limitation, which is strongly associated with increased chronic inflammatory responses to noxious particles or gases (mainly tobacco smoke) in the airways and lungs. Recently, several studies have shown that pulmonary hypertension and pulmonary vascular remodeling are the most important and critical links in the development of COPD in patients with pulmonary heart disease [5, 34, 35]. Therefore, controlling pulmonary vascular remodeling and pulmonary hypertension induced by COPD is important for delaying its cardiovascular complications. The previous study shows that hypoxia, inflammation, and other stimuli can cause the process of damage and repair of bronchi and pulmonary vessels occurs again and again. It will lead to pulmonary vascular remodeling if the progression gets worse [36–38]. CD30 is associated with inflammatory diseases, such as asthma. We speculated that CD30 and CD30L might also be associated with the occurrence and progression of COPD, even with the severity of COPD. CD30 is a member of tumor necrosis factor and nerve growth factor receptor superfamily. It is usually expressed in the production of Th2 cytokines CD4+T and CD8+T lymphocytes [19, 39]. sCD30 is released by activated human Th2 and Th0 lymphocyte. Studies have found that the level of sCD30 is elevated in Th2 related diseases including Omenns' syndrome [40] and systemic sclerosis. However, the level of sCD30 is reduced in Th1 related diseases such as multiple sclerosis and Crohn's disease [41, 42]. Additionally, serum sCD30 expression is increased in allergic patients. CD30 expression is positively correlated to disease development in allergic disease [43]. In addition, CD30 plays an important role in inducing eosinophil apoptosis [44]. The mechanisms of CD30-induced eosinophil apoptosis remain unclear. Some studies have found that high expression of CD30 activated NF-kB in Hodgkin lymphoma. Pizzolo et al. found that the level of serum sCD30 in patients with HIV is paralleled with the high replication of HIV in vivo [45]. Therefore, CD30 is considered to be a unique marker for Th2 lymphocytes, the critical cell in the allergic inflammation. However other studies found that CD30 was also highly expressed in patients with bronchial asthma, but not positively related to asthma severity [46]. CD30 is associated with the severity of inflammatory diseases such as inflammatory bowel disease, allergic bronchitis, rheumatoid arthritis, and asthma [47–49].

Our study results showed levels of CD30 and CD30L in both serum and tissue were significantly elevated, indicating that CD30 was an inflammatory factor involved in the formation of COPD. CD30L binding CD30 induced cytokine production by activating the signaling pathways involved in cell proliferation and differentiation [23]. We showed that both CD30 and CD30L were significantly decreased by anti-CD30 antibody in serum and tissue. Moreover, anti-CD30 antibody decreased MMP-9 and VEGF expression in tissue and improved structural pulmonary vascular remodeling. It was further indicated that CD30 was the inflammatory factor in the course of COPD genesis and development.

Our results showed that the rat pulmonary arteries in COPD rats were significantly thickened compared with normal rats. In addition, the lumen area was also significantly reduced in COPD rats, whereas WT and WA% were significantly increased. This suggested that thickened vascular wall, lumen stenosis, and remodeling in the pulmonary arterioles were obvious in COPD rats. Moreover, we showed that anti-CD30 antibody obviously decreased the 50–90 um and 90–200 *μ*m vascular remodeling indexes of WT and WA% in COPD rats. The results show that the anti-CD30 antibody intervention changed pulmonary vascular structure and changed 50–90 um vascular remodeling index from constrictive remodeling to expansive remodeling, known as compensatory remodeling. Contractile remodeling was not conducive to the progress of the disease, whereas the expansion of remodeling was conducive to disease control or relief. The above results suggested that CD30 affected the structural remodeling of pulmonary arterioles and that anti-CD30 antibodies might play a protective role in remodeling the structure of pulmonary arterioles.

Studies have shown that hypoxia induces upregulation of VEGF expression in human pulmonary artery smooth muscle cells by protein kinase C activated NF-kappa B [50]. VEGF can also cause the migration of vascular smooth muscle cells by activating the ROS signal and NF-kappa B, suggesting that the expression of VEGF is related to pulmonary vascular remodeling [51]. In this study, the expression of VEGF in lung tissue in COPD group was increased significantly. Anti-CD30 antibody reduced the expression of VEGF in COPD rats. Moreover, CD30 and CD30L were positively correlated with VEGF in COPD rats. This suggested, in pulmonary artery remodeling, CD30 and CD30L as the inflammatory factor were involved in stimulating VEGF secretion, but the specific mechanism was unknown. MMP-9 is a factor promoting the expression of VEGF. In this study, the expression of MMP-9 in lung tissue was significantly increased in COPD group. The anti-CD30 antibody decreased MMP-9 expression in COPD rats. We showed there was a positive correlation among CD30, CD30L, MMP-9, and VEGF, suggesting that CD30 and CD30L as inflammatory factors induced MMP-9 to promote VEGF. Finally, at the cellular level, we showed that the survival rate of HPAEC and HPASMC was decreased, cell migration ability and apoptosis rate were increased, and remodeling related indexes ECM1 and MMP-2 were decreased under hypoxic conditions. The recombinant CD30 protein under the intervention can improve the survival and migration inhibition of hypoxic cells and decrease the apoptosis rate of hypoxia cells and the expression of MMP-2 in concentration-dependent manner, but no significant effect on ECM1 and MMP-9. Inflammatory factor CD30 may influence the HPAEC and HPASMC cell growth and migration and inhibited hypoxia-induced apoptosis of HPASMC and HPAEC. Additionally, CD30 regulated MMP-2 expression to promote extracellular matrix formation, thus affecting the pulmonary vascular remodeling.

Our study showed that the CD30 and CD30L were associated with pulmonary vascular remodeling and inflammatory in COPD and participated in the pulmonary vascular remodeling and inflammation. Therefore, CD30 and CD30L can be used as a marker of inflammation of COPD, an indicator of vascular remodeling. The pathogenesis of COPD pulmonary vascular remodeling is very complex, involving a large number of cytokines. This study only detected the MMP-9, MMP-2, VEGF, and ECM1 levels. It has been confirmed that several other indicators have effect on vascular remodeling. This needs to be under studied further in a future study. Notably, part of the results were not consistent between in vivo and in vitro. For example, MMP-9 was not significantly changed in the two hypoxic cells, but the expression was obviously upregulated in COPD rats. This raises the possibility that upregulation of MMP-9 was involved in a mutual interaction between different types of cells in vivo.

## Figures and Tables

**Table 1:** Clinical data of participants.

Index	Groups		*P* value
	AECOPD	Stable COPD	
Age	75.00 ± 7.60	70.05 ± 8.84	0.006
Heart rate (minute)	92.00 ± 15.38	78.12 ± 12.49	<0.0001
Smoke (packs.year)	57.00 ± 109.29	16.55 ± 22.86	0.021
Oxygen saturation (%)	94.02 ± 7.01	92.88 ± 9.23	0.516
PaO_2_ (mmHg)	82.62 ± 22.73	82.69 ± 23.62	0.989
PaCO_2_ (mmHg)	50.23 ± 14.63	40.74 ± 7.67	<0.0001
PaO_2_/FiO_2_ (mmHg)	320.24 ± 109.63	366.74 ± 139.91	0.087
FEV1 (L)	0.77 ± 0.41	1.37 ± 0.50	<0.0001
FEV1/FVC (%)	44.90 ± 13.02	58.20 ± 10.45	<0.0001
LVEF (%)	64.98 ± 8.61	62.50 ± 16.83	0.389
PASP (mmHg)	34.11 ± 14.26	26.21 ± 9.19	0.003
FiO_2_ (%)	27.29 ± 7.12	25.81 ± 11.51	0.470
FEV1% (%)	36.12 ± 13.69	58.12 ± 18.37	<0.0001

**Table 2:** WA% and WT value in rat model lung tissue.

Index	0–50 *µ*m			50–90 *µ*m			90–200 *µ*m		
	CTR	COPD	Anti-CD30 COPD	CTR	COPD	Anti-CD30 COPD	CTR	COPD	Anti-CD30 COPD
WA%	0.56 ± 0.08^*∗*#^	0.65 ± 0.14	0.65 ± 0.29	0.59 ± 0.11^*∗*#^	0.65 ± 0.14^△^	0.37 ± 0.12	0.46 ± 0.18^*∗*^	0.67 ± 0.17^△^	0.31 ± 0.12
WT	6.96 ± 1.93^*∗*#^	7.34 ± 2.45	7.63 ± 2.46	11.88 ± 3.34^*∗*#^	13.76 ± 4.64^△^	7.17 ± 2.58	14.57 ± 6.89^*∗*^	30.21 ± 13.10^△^	13.42 ± 7.85

^**∗**^CTR group verus COPD group, *P* < 0.05. ^#^CTR group versus anti-CD30 COPD group. ^△^COPD group versus anti-CD30 group,  *P* < 0.05.
